# How’s Your Sugar? Evaluation of a Website for Aboriginal People With Diabetes

**DOI:** 10.2196/diabetes.6930

**Published:** 2017-04-04

**Authors:** Karen Adams, Anna Liebzeit, Jennifer Browne, Petah Atkinson

**Affiliations:** 1 Gukwonderuk Faculty of Medicine Nursing and Health Science Monash University Clayton Australia; 2 Monash University Clayton Australia; 3 Victorian Aboriginal Community Controlled Health Organisation Collingwood Australia

**Keywords:** Aboriginal and Torres Strait Islander peoples, type 2 diabetes mellitus, Indigenous populations, Internet

## Abstract

**Background:**

Australia’s Aboriginal and Torres Strait Islander peoples (hereafter referred to as “Aboriginal people”) have the longest continuing culture in the world, living sustainably for at least 65,000 years on the Australian continent. In relatively recent times, colonization processes have resulted in Aboriginal people experiencing unacceptable health inequalities compared with other Australians. One disease introduced due to colonization is diabetes, the second leading cause of death for Aboriginal peoples.

**Objectives:**

The objective of this study was to describe the construction and utilization of the website “How’s Your Sugar, ” a website for Aboriginal people with type 2 diabetes (herein after referred to as diabetes). The questions for the evaluation were as follows: how was the website constructed; did target groups utilize the website; and did engagement with the website improve diabetes management.

**Methods:**

A mixed-method study design was employed. A content analysis of project documents provided information about the website construction. Data from Google analytics provided information about website utilization. To describe patterns of website sessions, percentages and numbers were calculated. A voluntary survey provided more information on website utilization and diabetes self-management. Percentage, numbers, and 95% CIs were calculated for each variable. A chi-square test was performed for Aboriginal status, age, gender, and Aboriginal diabetic status using Australian population estimates and Aboriginal diabetes rates.

**Results:**

The website development drew on Aboriginal health, social marketing, interactive health promotion frameworks, as well as evidence for diabetes self-management. The website build involved a multidisciplinary team and participation of Aboriginal diabetics, Aboriginal diabetic family members, and Aboriginal health workers. This participation allowed for inclusion of Aboriginal ways of knowing and being. The highest number of website sessions came from Australia, 98.15% (47,717/48,617) and within Australia, Victoria 50.97% (24,323/47,717). There were 129 survey respondents, and the distribution had more female, 82.9% (107/129, 95% CI 76-88), Aboriginal, 21.7% (28/129, 95% CI 16-30), and Aboriginal diabetic, 48% (13/27, 95% CI 31-66) respondents than expected with *P*<.001 for these three groups. Most common reasons for visits were university assignment research, 40.6% (41/101), and health workers looking for information, 20.8% (21/101). The sample size was too small to calculate diabetes self-management change.

**Conclusions:**

Inclusion of Aboriginal ways of knowing and being alongside other theoretical and evidence models in Web design is possible. Aboriginal people do utilize Web-based health promotion, and further understanding about reaching to this population would be of use. Provision of an education resource would likely have enhanced educational engagement. Web-based technologies are rapidly evolving, and these can potentially measure behavior change in engaging ways that also have benefits for the participant. A challenge for designers is inclusivity of cultural diversity for self-determination.

## Introduction

Australia’s Aboriginal and Torres Strait Islander peoples (hereafter referred to as “Aboriginal people”) have the longest continuing culture in the world, living sustainably for at least 65,000 years on the Australian continent [1]. In relatively recent times (the last 230 years), the process of colonization has resulted in Aboriginal people experiencing unacceptable health inequalities compared with other Australians. Almost 10 years have passed since Australian governments agreed to closing the gap and improving Aboriginal health outcomes [2]. However, only minimal progress has been made. The median age at death for Aboriginal people is 24 years lower than that for non-Aboriginal Australians [3]. Despite this, there is a policy commitment to achieve equality in health status and life expectancy by 2031 [4].

One disease that colonization introduced to Aboriginal people is diabetes, the second leading cause of death among the Aboriginal population [5]. National surveys have estimated that approximately 1 in 10 Aboriginal people have diabetes, which is more than 3 times higher than that in the non-Aboriginal population [6,7]. In the 25- to 34-year age group, this prevalence is 5 times higher, indicating the earlier development of diabetes in the Aboriginal population [6]. Earlier onset of diabetes increases the chance of developing complications such as cardiovascular disease, renal failure, and retinopathy later in life [8]. Appropriate and effective diabetes management is essential for reducing morbidity and mortality among Aboriginal people.

Education and self-management are important components of diabetes care [9]. There is evidence that culturally appropriate self-management programs led by Aboriginal peers can be effective at improving participants’ self-management practices, physical activity levels, and quality of life [10]. Professional- and nonprofessional-led Web-based programs for diabetics have been shown to provide improvement [11]. The recently released Australian National Diabetes Strategy [12], which includes a specific goal of reducing the impact of diabetes among Aboriginal people, calls for expansion of consumer engagement and self-management initiatives. One recommendation is that peer support programs (either face-to-face, telephonic, or Web-based) are accessible to all with diabetes [13].

Health promotion aimed at Aboriginal people is increasingly focusing on Web-based and digital environments [14]. These tools come in a range of formats with theory underpinning construction varied and often not described [14]. However, these tools do have potential to influence health behaviors. Web-based interventions for diabetes can improve general knowledge, and tracking and monitoring of diabetes, however, have not been shown to improve depression or anxiety. In addition, theory-based Web designs are more effective for behavior change [11]. The types of Web-based tools available for people living with diabetes are evolving rapidly and now combine elements of patient records, interactivity, feedback loops, and multiple hardware devices, such as phone, glucometer, tablet, and computer [15].

Many Aboriginal people access the Internet. In the 2011 Census, 63% of Aboriginal households reported having Internet connection, up from 40% in 2006. The quality of connection was high with 85% most frequently connected using broadband, 11% other types of connections (eg, mobile phones), and 4% dial-up connections. Younger Aboriginal people (aged 24 years and less) were more likely to have home Internet connection than those aged 55 years and more (63% and 42%, respectively) [16].

Sparse data exist about Aboriginal engagement in various websites [13]. Information about end user experiences with the technologies is also rare. One study in Victoria, Australia, explored young women’s research recruitment from social networking sites with an Aboriginal participant rate consistent with Aboriginal population proportion [17]. Another study of Aboriginal people’s Facebook use found that Aboriginal people were engaging with the platform to actively express identity and network with other Aboriginal people [18]. This study aimed to describe the construction and utilization of the website “How’s Your Sugar,” a website for Aboriginal people with type 2 diabetes (herein after referred to as diabetes). The questions for the evaluation were as follows: how was the website constructed; did target groups utilize the website; and did engagement with the website improve diabetes management.

## Methods

### Study Design

The How’s Your Sugar website was developed during October 2009 to May 2010, and launched in June 2010. The website build was supported by a 12-month grant from the Australian Department of Health and Aging. Ethics approval for the evaluation was obtained from the Victoria University Human Research Ethics Committee. The evaluation included creation of a Logframe Matrix [19] (Table 1), and mixed methods were used to collate evaluation data including project document content analysis, website data collection, and survey.

**Table 1:** Logframe matrix.

Objectives	Indicator	Data source	Assumption
Goal: Develop a culturally relevant and evidence-based website to support Aboriginal people with type 2 diabetes to manage well-being.	Was an evidence and theory base used to develop the website?	Program documents	Team has the skills required to build the website
	Was cultural relevance included in the website?		
Outcome: To support Aboriginal people with type 2 diabetes to manage well-being	Did the website focus on managing type 2 diabetes?	Program documents	A website can support people with type 2 diabetes to manage well-being
	Did the resource support Aboriginal people with type 2 diabetes?	Website content	
		Web-based survey	
Outputs: Website supports Aboriginal people with type 2 diabetes to manage well-being	Was the website utilized by the target group? For instance, Aboriginal people with diabetes, friends and family members of Aboriginal people with diabetes, and health workers?	Website analytics	Aboriginal people with type 2 diabetes will engage with the website Health professionals will access the website
		Web-based Survey	
Activities: Website is accessible, promoted, and utilized	Was the website accessed?	Google analytics	Aboriginal people with type 2 diabetes engaging with the website will improve management of diabetes and well-being Health professionals will learn about Aboriginal people and staying well with diabetes
	Was the website promoted?	Web-based survey	
	Did engagement occur with the website?		

### Website Build

Data about the website build were extracted from documents generated by the project that described elements of the website build process. These project documents included minutes of project team meetings (6), reports to funding bodies (4), project planning session notes (8), communication strategy (1), conference presentations (2) and written notes taken at social marketing interviews (6), and a focus group (1). The documents were reviewed using content analysis with the aim to address the following logframe matrix indicators: was an evidence and theory base used, was cultural relevance included, did the website focus on diabetes management, and was the website promoted.

### Website Utilization

To understand more about website access and usage, two sources of data were collected using Google Analytics [20] and SurveyMonkey (SurveyMonkey Inc) [21]. Google Analytics provided data on website sessions this data collection addressed the logframe matrix indicator questions: was the website accessed and did engagement occur with the website. To describe patterns of website sessions, percentages and numbers were calculated.

**Figure 1:**
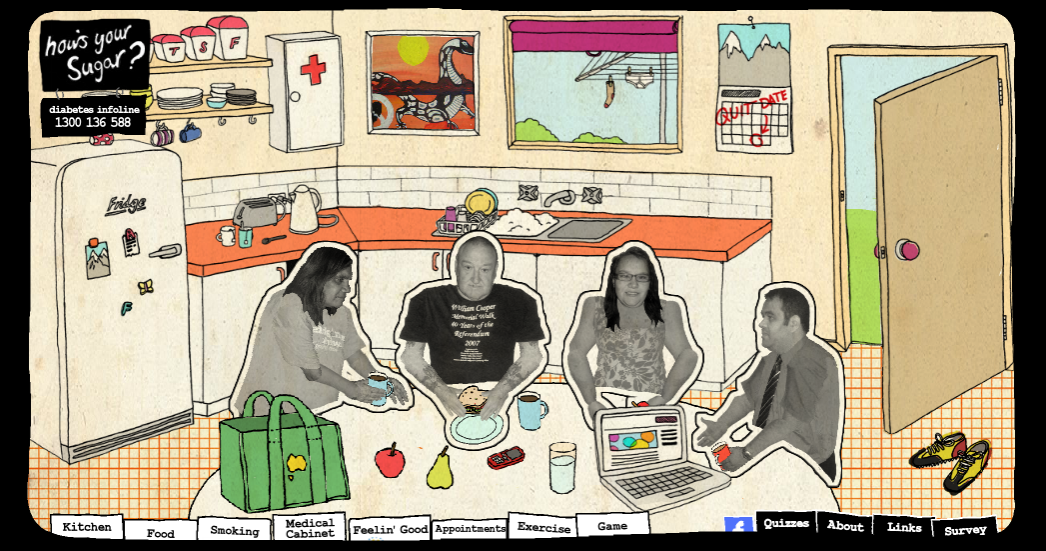
Opening page of the website.

A voluntary survey was developed using SurveyMonkey with a link to this on the website front page (Figure 1). The survey allowed only 1 response per respondent through IP address tracking. The survey addressed the logframe matrix indicator questions: did the resource support Aboriginal people with type 2 diabetes, was the website used by the target group, and did engagement occur with the website. The survey included an explanatory statement, which then led to the survey questions. The first survey page asked for age, gender, reason for visit, and Aboriginal status. Those affirming Aboriginal status went to a second page that asked for diabetic status. Those indicating they were diabetic went to a fourth page asking questions about diabetes management with questions relating to the 10 steps for living well with diabetes. These respondents were invited to provide an email to be asked further questions in a 6-month time period. Further details on these variables can be found in Table 2. Percentage, numbers, and 95% CIs were calculated for each variable. A chi-square test was performed for Aboriginal status, age, gender, and Aboriginal diabetic status using Australian population [22] and Aboriginal diabetes estimates [6-7]. Calculations were conducted in the Microsoft Excel version 14.4.2 (Microsoft Corporation) [23].

## Results

### Website Build

The How’s Your Sugar project team was based at Victoria University in Melbourne and consisted of an Aboriginal nurse and two Aboriginal educators experienced in adult education. The project team also drew on expertise from the Victorian Aboriginal Community Controlled Health Organization and an Aboriginal worker from Diabetes Victoria. Initially the team reviewed websites aiming to provide information to Indigenous people with diabetes in Australia, New Zealand, Canada, and the United States. These commonly featured static materials, such as written text, images, and downloadable PDF information. Imagery accompanying the writing commonly included a mix of Indigenous iconography and clinical elements, such as a health professional in a white coat with a stethoscope. The materials provided didactic information on what a person should do to manage their diabetes, and were written in simple English from the perspective of a non-Aboriginal health professional as expert speaking to a client. One project team member stated during this review of websites, “Who do they think they are talking to? It’s like they stuck some Aboriginal artwork on it and said ‘that’ll do’, its patronizing.” The project team recognized the potential for greater use of engaging and interactive website elements that could be inclusive of adult learning pedagogy and Aboriginal ways of knowing and being [24]. Aboriginal websites reflecting this type of inclusion, such as 12canoes [25] and Mission Voices [26] influenced the How’s Your Sugar construction.

The team identified the 10 steps for living well with diabetes as a practical and evidence-based health education message for the website target groups. The 10 steps recommended 30 minutes of daily physical activity, taking prescribed medications, regularly checking blood sugar levels, visiting a general practitioner regularly, eating fruits and vegetables, having an annual foot and eye check, ceasing smoking, drinking alcohol in moderation, and living well [27]. Three frameworks informed the How’s Your Sugar website construction, including interactive health communication [28], social marketing [29], and the definition of Aboriginal health [4]. Multimedia Appendix 1 provides further details about the application of the 3 frameworks.

The project team conducted interviews, and focus groups were the target groups of 4 Aboriginal people with diabetes (2 males and 2 females), 2 Aboriginal health workers (1 male and 1 female), 4 mature Aboriginal Victoria University students with family or friends with diabetes, and 4 employees at Victoria University with family or friends with diabetes. Travel to conduct broader consultation was not possible due to budget restriction. The 4 people with diabetes were individually asked about the types of information they had found helpful for managing their diabetes. These people described a common experience of health professionals providing information about what to do to manage diabetes and learning from peers with lived experiences of managing diabetes more about how to manage diabetes. For instance, 1 person described, “The doctor told me what I had to do and gave me the pamphlets but it wasn’t until I was yarning up to someone like me that I really understood what I needed to do and how I could do it.”

The cultural importance of managing diabetes in relation to social connection for Aboriginal people has been highlighted in other studies [30]. The peers described receiving this “how to” information in informal environments and liked being able to laugh and joke about their diabetes to relieve stress. For example, 1 person said “You feel like you need to be doing the right thing all the time, it’s good to just have a laugh about it.” Use of humor by Aboriginal people as a well-being mechanism to relieve stress and talk about uncomfortable areas has been described elsewhere [31]. When asked about Web-based interactions, people said they regularly accessed email, social media, Web-based video and games. Based on this information, draft designs were made for further review. At this stage, the project team also began to investigate a suitable website designer to work with. Abilities to be flexible, to be transparent, and listen carefully to the project team needs were noted as desired qualities, which led to the engagement of a suited designer.

A set of draft designs were reviewed by the same 4 people with diabetes and the Aboriginal health workers. All were asked individually to reflect on what they liked, what they did not like, and what they would change. The focus group was asked the same questions. Participants collectively recommended the following: incorporation of more entertainment and humor, storytelling, cloud symbolism;, a barbecue in the backyard, inclusion of Koorie English [32], removing a no-smoking symbol from the website area with videos about tobacco cessation so smokers would not be repelled, providing something that welcomed people verbally to the site, inclusion of other fun health promotion activities aimed at Aboriginal people, inclusion of caring for country, and ensuring that men and women were represented in the site as peers and Aboriginal health workers. This advice was incorporated into the final design. During development, a non-Aboriginal health professional urged the project team to include a diabetes educator on the website to ensure that the messages conveyed were accurate. The project team thought that this could diminish the Aboriginal health worker and peer roles; however, a diabetes support and smoking cessation call line was included in case website visitors were seeking further information.

**Figure 2:**
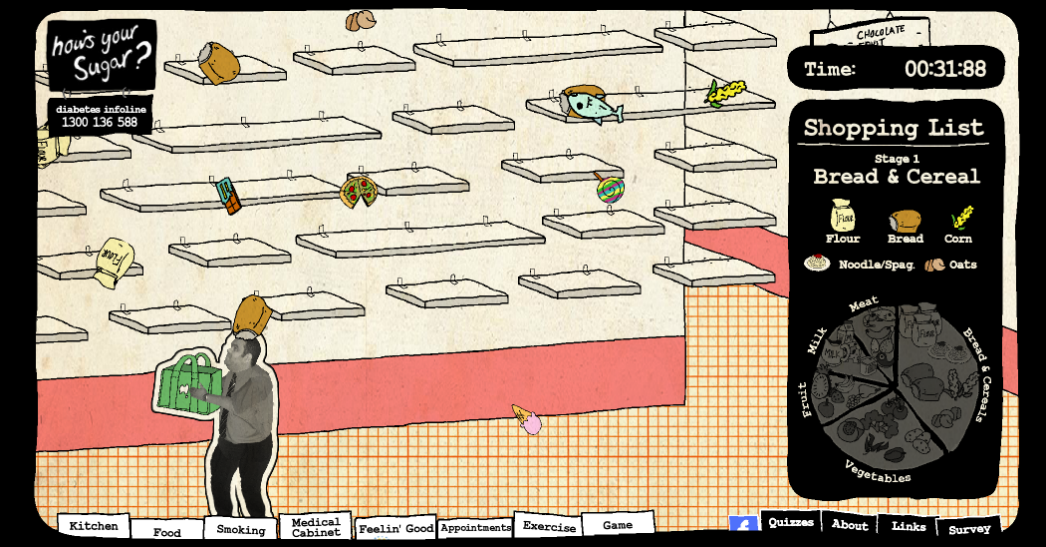
Nutrition game.

The name How’s Your Sugar? was suggested by 1 of the Aboriginal health workers, as it is a common way for Aboriginal people to ask a person with diabetes how they are feeling or travelling with their diabetes. The final design of the website provided an opening page of a kitchen, a familiar informal space where people gather to yarn or talk replicating a generalized version of what it is like for an Aboriginal person to visit a family member or friend’s home. Four peers gathered around the table inviting visitors to click on something. Visitors could navigate around the space and interact with various elements in the website, finding their own pathway through the material. Each peer featured in short videos about how they managed their diabetes in regard to the 10 steps for living well with diabetes. In addition, short videos of 4 Aboriginal health workers discussing self-care and 6 people reflecting on smoking cessation were included. All of the short videos were placed in a relevant space in the website. For instance, videos about diet were placed in the refrigerator and visitors clicked on the refrigerator door to view them. A nutrition game (Figure 2), quiz, and email reminder about recommended minimum appointments were also integrated into the website. Text message reminders were also considered; however, the cost of doing this was beyond the project budget. More details about the design can be viewed in Multimedia Appendix 1.

The project team developed a communication strategy to promote the website. This included posting packages of 50 to 100 fridge magnets to each of the Aboriginal Community Controlled Health Services listed as members of the National Aboriginal Community Controlled Health Organization. The project team wrote an article for the Aboriginal and Torres Strait Islander Health Worker Journal [33], and referral links were negotiated with the Indigenous Healthinfonet, the Victorian Aboriginal Community Controlled Health Organization, and Diabetes Victoria websites. An unexpected outcome was that the How’s Your Sugar website was also promoted by other websites [34-37].

### Website Utilization

**Figure 3:**
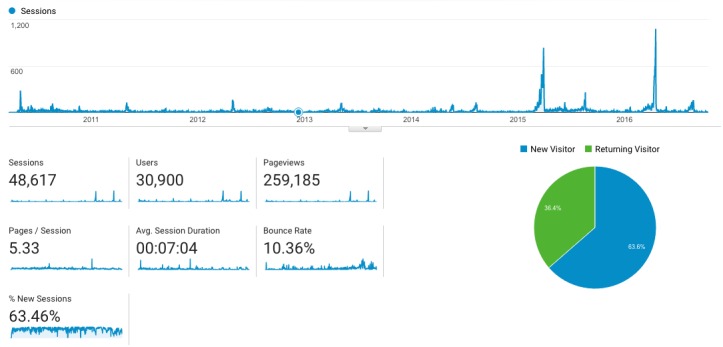
Website session data.

Data from Google Analytics (Figure 3) revealed a total of 48,617 sessions from April 2010 to October 2016, with 30,852 out of 48,617 (64%) new sessions. Mean session duration and mean pages viewed per session suggested a pattern of engagement with the website. There was an annual pattern of increased sessions during the months April to May and August to September. In 2015 and 2016, there were spikes indicating increased site usage. The majority of sessions originated from Australia, 98.15% (47,717/48,617) with the highest number of sessions in Victoria 50.97% (24,323/47,717). Sources of website access included search engines and referring websites. Operating systems used per session indicated desktop preference.

For the same time frame, there were 129 respondents to the survey (Table 2) with 30,700 individual users of the website. This indicated a less than 1% response rate, not unusual for voluntary Web-based surveys [38]. The survey respondent distribution had more females, Aboriginal people, and Aboriginal people with diabetes than expected with *P*<.001. Age group distribution of respondents differed from that of the Australian population, with the 26- to 35-year age group highest, followed by the 46- to 55-year age group. The most common reasons for visiting the site included research for a university assignment and health workers looking for information. Eleven Aboriginal respondents answered questions about self-assessed management of diabetes. This number was too small to calculate statistical significance lacking type II power; however, the data are provided in Table 2 for possible meta-analysis. Of the 11 respondents, 6 agreed to be resurveyed for diabetes self-management, with this number also too small to calculate statistical difference.

**Table 2:** Characteristics of survey respondents.

Characteristics		n (%)	95% CI	*P* value
**Age group (n=127)**				<.001
	18-25	29 (22.8)	14-27	
	26-35	44 (34.6)	23-38	
	36-45	23 (18.1)	11-23	
	46-55	30 (23.6)	15-28	
	56-65	8 (6.3)	3-10	
	66+	3 (2.4)	0.7-6	
**Gender (n=129)**	Female	107 (82.9)	76-88	<.001
	Male	22 (17.1)	12-25	
**Reason for site visit (n=101)**				
	Research for university assignment	41 (40.6)	32-50	
	Health worker looking for information	21 (20.8)	14-30	
	Curiosity	18 (17.8)	12-26	
	Diabetic looking for information	12 (11.8)	7-20	
	Research	8 (7.9)	4-15	
	Person with diabetic family member looking for information	1 (9.9)	0.2-5	
**Aboriginal status (n=129)**				<.001
	Aboriginal	28 (21.7)	16-30	
	Non-Aboriginal	101 (78.3)	70-85	
**Aboriginal diabetic status (n=27)**				<.001
	Diabetic	13 (48)	31-66	
	Non-Diabetic	14 (52)	34-69	
Aboriginal diabetic self-management (n=11)				
**Regularly monitors blood sugar**				
	Never	2 (18)	5-48	
	Rarely	2 (18)	5-48	
	Sometimes	3 (27)	10-57	
	Mostly	1 (27)	2-37	
	Always	3 (27)	10-57	
**Takes medication regularly**				
	Never	2 (18)	5-48	
	Rarely	0 (0)	-	
	Sometimes	2 (18)	5-48	
	Mostly	5 (46)	21-72	
	Always	2 (18)	5-48	
**Recommended diabetic diet**				
	Never	2 (18)	5-48	
	Rarely	3 (27)	10-57	
	Sometimes	0 (0)	-	
	Mostly	5 (46)	21-72	
	Always	1 (9)	2-37	
**Recommended exercise**				
	Never	2 (18)	5-48	
	Rarely	3 (27)	10-57	
	Sometimes	2 (18)	5-48	
	Mostly	0 (0)	-	
	Always	4 (36)	16-65	
**Checks feet daily**				
	No	6 (55)	28-79	
	Yes	5 (46)	21-72	
**Visit general practitioner at least twice a year**				
	No	5 (46)	21-72	
	Yes	6 (55)	28-79	
**Eyes checked in last 2 years**				
	No	6 (55)	28-79	
	Yes	5 (46)	21-72	

## Discussion

### Principal Findings

The How’s Your Sugar website development drew on Aboriginal health, social marketing, and interactive health promotion frameworks. An evidence base for diabetes self-management also informed the website content. The website build involved a multidisciplinary team and participation of Aboriginal diabetics, Aboriginal diabetic family members, and Aboriginal health workers. This participation allowed for inclusion of Aboriginal ways of knowing and being [27] through soundscape, imagery, and interaction. This was important, as approaches to dealing with Aboriginal health often omit Aboriginal knowledge and focus instead on the illness and pathology that has arisen from cultural dispossession. This can effectively reduce self-determination of Aboriginal people by imposing a western cultural model not inclusive of Aboriginal well-being modalities, contributing further to identity loss and dislocation [39]. Rather than simply considering that Aboriginal people have a right to diabetes education, How’s Your Sugar embedded this in Aboriginal ways of knowing and being. This is part of a global political movement in self-determination that aims to include indigenous knowledge and cultural practices in the everyday life [40]. To some extent this positioning is consistent with the self-determining component of the Chronic Care Model, whereby those experiencing chronic conditions ideally should feel empowered to maintain well-being [41]. Other authors have also called for those working in health to question how this locus of control relates to Aboriginal peoples [42].

The highest number of website sessions came from Victoria, the geographic region where the website was created. This might indicate increased relevancy for people living in this area. Varying promotion of How’s Your Sugar in different states and territories might have also influenced this pattern. The How’s Your Sugar website was successful in attracting visitors via referring websites, and this type of promotion could be considered by others. Desktop access was preferred. However, How’s Your Sugar was constructed at a time when mobile and tablet Internet use was emerging and so the content does not translate well to these, thus likely impacting on findings. Since inception of the website, there has been much technology change [15] and if How’s Your Sugar were made today, different Web-based tools would be available.

The study survey respondent distribution had more Aboriginal and Aboriginal people with diabetes than expected. It is possible that these people were more motivated to respond; however, it does indicate that this target group was accessing the website. Survey respondent distribution had more females than expected, and it is unclear why. Reasons for visiting the website varied, including personal diabetes education, university assignments, research, and health workers seeking information. This indicates that the target group of health workers were also accessing the website. The spikes in annual website sessions are indicative of Australian teaching semesters, and this may explain these spikes, particularly as the survey data indicate that university students were accessing the site and that the website was promoted by tertiary education institutions. The increased spikes in usage may indicate that more students were using the site over the past 2 years. Development of an associated education resource may have enhanced engagement with this group.

### Limitations

The survey was unable to describe diabetes management behavior change due to limited numbers of respondents. In Aboriginal research, gaining enough numbers of people for statistical analysis is often a challenge [43]. We suggest to others attempting this type of research to consider other means of measuring change, and technology developed since the inception of this website could facilitate this. The survey is also limited to a small response rate, and the data represent those willing to undertake the survey and so is not representative of all visitors to the website.

### Conclusions

This study identifies that Aboriginal ways of knowing and being can be included in website designs alongside other theoretical and evidence models. It also highlights that Aboriginal people do engage with Web-based health promotion and further understanding about reaching to this population would be of use. The website was also being utilized for education purposes, and provision of an education resource would likely have enhanced this engagement. Web-based technologies are rapidly evolving, and these can potentially measure behavior change in engaging ways that have benefits for the participant. A challenge for designers is inclusivity of cultural diversity for self-determination.

## Appendix

Multimedia Appendix 1 Application of frameworks.
