# Supplementary material for: How’s Your Sugar? Evaluation of a Website for Aboriginal People With Diabetes
Source: JMIR Diabetes. 2017 Apr 4;2(1):e6. doi: 10.2196/diabetes.6930 (PMC6238832; doi:10.2196/diabetes.6930)
Supplement: Multimedia Appendix 1 [file diabetes_v2i1e6_app1.pdf]

Framework applications for How's Your Sugar

Interactive Health Promotion [1]

Elements

- *Peer Information Exchange*
- *Informed decision making*
- *Managing Health Service Demand*
- *Self-care promotion*
- *Health Promotion Behaviour*
- *Information relay*

Aboriginal health definition [2]

Elements

- *Community*
- Cyclic concepts: The website is cyclic with no beginning or no end. Visitors can navigate their own way around the website finding their own path.
- *Cultural wellbeing*
- *Social well-being*
- *Emotional wellbeing*

Social Marketing [3]

Elements

- *Exchange*
- *Behaviour change*
- *Competition*
- *Marketing mix*
- *Consumer research*
- *Segmenting and targeting*

References

1. Eng, T., et al., Introduction to evaluation of interactive health communication applications. Science panel on interactive communication and health. American Journal of Preventative Medicine, 1999. 16(1): p. 10-15. PMID: 9894549
2. Australian Government. National Aboriginal and Torres Strait Islander Health Plan 2013–2023, 2013. ISBN: 978-1-74241-980-0
3. Lee, N. and P. Kofler, Social marketing : influencing behaviors for good. 2011, Thousand Oaks, California SAGE Publications ISBN-10: 1412981492

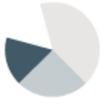

Framework applications for How's Your Sugar

Interactive Health Promotion [1]

Elements

- *Peer Information Exchange*
- *Informed decision making*
- *Managing Health Service Demand*
- *Self-care promotion*
- *Health Promotion Behaviour*
- *Information relay*

Aboriginal health definition [2]

Elements

- *Community*
- *Cyclic concepts*
- *Cultural wellbeing*
- *Social well-being*
- *Emotional wellbeing*

Social Marketing [3]

Elements

- *Exchange*
- *Behaviour change*
- *Competition*
- *Marketing mix*
- *Consumer research*
- *Segmenting and targeting*

References

1. Eng, T., et al., Introduction to evaluation of interactive health communication applications. Science panel on interactive communication and health. American Journal of Preventative Medicine, 1999. 16(1): p. 10-15. PMID: 9894549
2. Australian Government. National Aboriginal and Torres Strait Islander Health Plan 2013–2023, 2013. ISBN: 978-1-74241-980-0
3. Lee, N. and P. Kofler, Social marketing : influencing behaviors for good. 2011, Thousand Oaks, California SAGE Publications ISBN-10: 1412981492

Interactive Health Promotion [1]

Elements

Peer Information Exchange

Information is exchanged via four peers of diverse age and gender. In addition Aboriginal Health Workers who have a dual role as health providers and peers also provide information about staying well. A game and interactive elements also relay this information.

Informed decision making

Peers and Aboriginal Health Workers explain through narrative storytelling a diverse range of ways they experience the ten steps for living well with diabetes to assist inform decision-making. The nutrition game and other interactive elements promote informed choices.

Managing Health Service Demand

Visitors to the website are invited to enroll in email reminder for the minimum recommended health service visits; two annual GP appointments, an annual eye check and podiatry check. Phone numbers for diabetes and smoking cessation support are provided.

Self-care promotion

The peers discuss a range of ways in which they navigate self care representing the diversity of ways in which people can go about managing their life. The peers reflect on a variety of circumstances they encounter and problem solve. These stories are reinforced by interactive activities, such as, the shopping game.

Health Promotion Behaviour

The website reinforces the recommended ten steps for living well with diabetes through the lens of Aboriginal ways of knowing and being

Information relay

Is delivered via the peers through narrative storytelling and is embedded in Aboriginal ways of knowing and being. Information is also relayed through interactivity and entertainment.

Self-care promotion

The peers discuss a range of ways in which they navigate self care representing the diversity of ways in which people can go about managing their life. The peers reflect on a variety of circumstances they encounter and problem solve. These stories are reinforced by interactive activities, such as, the shopping game.

Information relay

Is delivered via the peers through narrative storytelling and is embedded in Aboriginal ways of knowing and being. Information is also relayed through interactivity and entertainment.

Health Promotion Behaviour

The website reinforces the recommended ten steps for living well with diabetes through the lens of Aboriginal ways of knowing and being

Peer Information Exchange

Information is exchanged via four peers of diverse age and gender. In addition Aboriginal Health Workers who have a dual role as health providers and peers also provide information about staying well. A game and interactive elements also relay this information.

Informed decision making

Peers and Aboriginal Health Workers explain through narrative storytelling a diverse range of ways they experience the ten steps for living well with diabetes to assist inform decision-making. The nutrition game and other interactive elements promote informed choices.

Managing Health Service Demand

Visitors to the website are invited to enroll in email reminder for the minimum recommended health service visits; two annual GP appointments, an annual eye check and podiatry check. Phone numbers for diabetes and smoking cessation support are provided.

Aboriginal health definition [2]

Elements

Cyclic concepts

The website is cyclic with no beginning or no end. Visitors can navigate their own way around the website finding their own path.

Community

The peers and Aboriginal Health Workers represent a community of Aboriginal people with diabetes in the website. They describe how they interact with community particularly in regard to staying well with diabetes. Other examples of community reference are that Mary G provides a community radio show and Aboriginal Health Workers encourage people to join in local community wellbeing activities.

Cultural wellbeing

The site reinforces Aboriginal culture through sound, presence of the peers and the imagery in the website. For example, the peers share real life stories in their own language about how they navigate diabetes and staying well in a colonised world. Embedded in the website are many cultural references in the landscape, such as, survival day, Aboriginal flag coloured shoes, Snake Condoms, Mary G. song, clouds shaped as Australian animals and more.

Social well-being

The website provides a social space, a home where people yarn around the kitchen table and socialise in the backyard. Yarning is a social communication method that allows people deep connection. The website is designed with an aim to make people feel socially welcome with a warm colour palate, familiarity and opportunities to engage.

Whole of life view

Rather than being broken up into individual parts, the website begins as a whole picture of the broader social world of the home. Visitors are then invited to explore the nuances of this and delve deeper into particular areas always returning to the bigger picture of the whole, represented by the home.

Emotional wellbeing

The peers describe varying ways in which they stay well emotionally. For example, one pays attention when she is feeling down and stops to see who is around her and reflect on what is happening. Another peer describes noticing negative thought patterns and purposely changing these to positive ones. Peers describe enjoying gardening, spending time with family, being artists and walking dogs as activities that bring enjoyment into their lives.

Eleme

Social well-being

The website provides a social space, a home where people yarn around the kitchen table and socialise in the backyard. Yarning is a social communication method that allows people deep connection. The website is designed with an aim to make people feel socially welcome with a warm colour palate, familiarity and opportunities to engage.

Emoti

Emotional wellbeing

The peers describe varying ways in which they stay well emotionally. For example, one pays attention when she is feeling down and stops to see who is around her and reflect on what is happening. Another peer describes noticing negative thought patterns and purposely changing these to positive ones. Peers describe enjoying gardening, spending time with family, being artists and walking dogs as activities that bring enjoyment into their lives.

Whole of life view

Rather than being broken up into individual parts, the website begins as a whole picture of the broader social world of the home. Visitors are then invited to explore the nuances of this and delve deeper into particular areas always returning to the bigger picture of the whole, represented by the home.

Cultural wellbeing

The site reinforces Aboriginal culture through sound, presence of the peers and the imagery in the website. For example, the peers share real life stories in their own language about how they navigate diabetes and staying well in a colonised world. Embedded in the website are many cultural references in the landscape, such as, survival day, Aboriginal flag coloured shoes, Snake Condoms, Mary G, song, clouds shaped as Australian animals and more.

Community

The peers and Aboriginal Health Workers represent a community of Aboriginal people with diabetes in the website. They describe how they interact with community particularly in regard to staying well with diabetes. Other examples of community reference are that Mary G provides a community radio show and Aboriginal Health Workers encourage people to join in local community wellbeing activities.

Cyclic concepts

The website is cyclic with no beginning or no end. Visitors can navigate their own way around the website finding their own path.

[illegible] the peers share real life stories in their own language about how they navigate diabetes and staying well in a colonised world. Embedded in the website are many cultural references in the landscape, such as, survival day, Aboriginal flag coloured shoes, Snake Condoms, Mary G, song, clouds shaped as Australian animals and more.

Social Marketing [3]

Elements

Exchange

Peers and Aboriginal Health Workers use narrative storytelling about how to achieve the ten steps for living well with diabetes. Interactive activities (game and quiz) and email health care reminders reinforce the message.

Behaviour change

Peers provide narrative storytelling examples of how they have gone about changing their behaviour in regard to the ten steps for living well with diabetes. Interactive activities and health care reminders reinforce these messages.

Competition

Competition in Hows Your Sugar often occurs as peers navigate behaviour choices. For instance, peers are faced with managing competition for healthy and unhealthy behaviour. being offered alcohol or unhealthy food as temptations and having to navigate this often describing winning health gains in the long term rather than experiencing short term enjoyment of social acceptance.

Marketing mix

1. Product: ten steps for living well with diabetes.
2. Price: For changing behaviour to manage diabetes is voiced by the peers and Aboriginal Health Workers and mostly related to feeling well, being a role model for family and living longer.
3. Place: a virtual familiar home with accompanying sound-scape and warm palette. Videos were filmed in people's familiar environments at home or workplace.
4. Promotion – Fridge magnets, referring websites, article in Aboriginal Health Worker Journal.

Consumer research

Interviews with Aboriginal people with diabetes to inform a draft website design. Secondary interviews and focus groups with Aboriginal people with diabetes, Aboriginal Health Workers and Aboriginal people with friends of family with diabetes to inform refinement of website design.

Segmenting and targeting

Target group was urban Aboriginal people living with diabetes and their supporters (family, friends and carers, including health professionals). Segmenting included gender and age diversity.

Behaviour change

Peers provide narrative storytelling examples of how they have gone about changing their behaviour in regard to the ten steps for living well with diabetes. Interactive activities and health care reminders reinforce these messages.

Consumer research

Interviews with Aboriginal people with diabetes to inform a draft website design. Secondary interviews and focus groups with Aboriginal people with diabetes, Aboriginal Health Workers and Aboriginal people with friends of family with diabetes to inform refinement of website design.

Segmenting and targeting

Target group was urban Aboriginal people living with diabetes and their supporters (family, friends and carers, including health professionals). Segmenting included gender and age diversity.

Exchange

Peers and Aboriginal Health Workers use narrative storytelling about how to achieve the ten steps for living well with diabetes. Interactive activities (game and quiz) and email health care reminders reinforce the message.

Competition

Competition in Hows Your Sugar often occurs as peers navigate behaviour choices. For instance, peers are faced with managing competition for healthy and unhealthy behaviour. being offered alcohol or unhealthy food as temptations and having to navigate this often describing winning health gains in the long term rather than experiencing short term enjoyment of social acceptance.

Marketing mix

1. Product: ten steps for living well with diabetes.
2. Price: For changing behaviour to manage diabetes is voiced by the peers and Aboriginal Health Workers and mostly related to feeling well, being a role model for family and living longer.
3. Place: a virtual familiar home with accompanying sound-scape and warm palette. Videos were filmed in people's familiar environments at home or workplace.
4. Promotion – Fridge magnets, referring websites, article in Aboriginal Health Worker Journal.

Framework applications for How's Your Sugar

Interactive Health Promotion [1]

Elements

- *Peer Information Exchange* — [illegible]
- *Informed decision making* — [illegible]
- *Managing Health Service Demand* — [illegible]
- *Self care promotion* — [illegible]
- *Health Promotion Behaviour* — [illegible]
- *Information relay* — [illegible]

Aboriginal health definition [2]

Elements

- *Community* — [illegible]
- *Cyclic concepts* — [illegible]
- *Cultural wellbeing* — [illegible]
- *Social well-being* — [illegible]
- *Whole of life view* — [illegible]
- *Emotional wellbeing* — [illegible]

Social Marketing [3]

Elements

- *Exchange* — [illegible]
- *Behaviour change* — [illegible]
- *Competition* — [illegible]
- *Marketing mix* — [illegible]
- *Consumer research* — [illegible]
- *Segmenting and targeting* — [illegible]

References

1. Eng, T., et al., Introduction to evaluation of interactive health communication applications. Science panel on interactive communication and health. American Journal of Preventative Medicine, 1999. 16(1): p. 10-15. PMID: 9894549
2. Australian Government. National Aboriginal and Torres Strait Islander Health Plan 2013–2023, 2013. ISBN: 978-1-74241-980-0
3. Lee, N. and P. Kofler, Social marketing : influencing behaviors for good. 2011, Thousand Oaks, California SAGE Publications ISBN-10: 1412981492

References

1. Eng, T., et al., Introduction to evaluation of interactive health communication applications. Science panel on interactive communication and health. American Journal of Preventative Medicine, 1999. 16(1): p. 10-15. PMID: 9894549
2. Australian Government. National Aboriginal and Torres Strait Islander Health Plan 2013–2023, 2013. ISBN: 978-1-74241-980-0
3. Lee, N. and P. Kofler, Social marketing : influencing behaviors for good. 2011, Thousand Oaks, California SAGE Publications ISBN-10: 1412981492
